# Flower Mapping in Grasslands With Drones and Deep Learning

**DOI:** 10.3389/fpls.2021.774965

**Published:** 2022-02-09

**Authors:** Johannes Gallmann, Beatrice Schüpbach, Katja Jacot, Matthias Albrecht, Jonas Winizki, Norbert Kirchgessner, Helge Aasen

**Affiliations:** ^1^Department of Computer Science, ETH Zürich, Zurich, Switzerland; ^2^Agricultural Landscape and Biodiversity Group, Agroscope, Zurich, Switzerland; ^3^Department of Agricultural Science, ETH Zürich, Zurich, Switzerland; ^4^Remote Sensing Team, Division Agroecology and Environment, Agroscope, Zurich, Switzerland

**Keywords:** unmanned aerial vehicle (UAV), abundance mapping, faster R-CNN, object detection, aerial image, machine learning, remotely piloted aerial vehicles (RPAS), meadow

## Abstract

Manual assessment of flower abundance of different flowering plant species in grasslands is a time-consuming process. We present an automated approach to determine the flower abundance in grasslands from drone-based aerial images by using deep learning (Faster R-CNN) object detection approach, which was trained and evaluated on data from five flights at two sites. Our deep learning network was able to identify and classify individual flowers. The novel method allowed generating spatially explicit maps of flower abundance that met or exceeded the accuracy of the manual-count-data extrapolation method while being less labor intensive. The results were very good for some types of flowers, with precision and recall being close to or higher than 90%. Other flowers were detected poorly due to reasons such as lack of enough training data, appearance changes due to phenology, or flowers being too small to be reliably distinguishable on the aerial images. The method was able to give precise estimates of the abundance of many flowering plant species. In the future, the collection of more training data will allow better predictions for the flowers that are not well predicted yet. The developed pipeline can be applied to any sort of aerial object detection problem.

## 1. Introduction

The service done by pollinators in farmlands is estimated to value more than 150 billion euros a year worldwide (Gallai et al., 2009). Their declining numbers (Hallmann et al., 2017) motivate many ecologists to study their interplay with the environment. Such studies include the assessment of flower abundance and distribution, which is an extremely time-consuming task. At the same time, quantification of floral resources is an increasingly important topic in ecological research with implications for both theoretical and applied ecological issues (Benadi and Pauw, 2018; Bergamo et al., 2020; Biella et al., 2020; Fantinato et al., 2021).

Many remote sensing technologies exist to assess plant diversity (Wang and Gamon, 2019; Lausch et al., 2020). In the last 10 years, rapid developments in sensor technology and robotics have enhanced the capabilities of unmanned aerial vehicles (UAVs) (Anderson and Gaston, 2013; Pajares, 2015; Sanchez-Azofeifa et al., 2017; Aasen et al., 2018b). Today, it is both technologically possible and financially affordable to take ultra-high spatial resolution images of large areas (several deka-hectares with a ground resolution of 1 cm per pixel). When UAVs are flying at a lower height and slower speed, even resolutions of down to millimeters per pixel can be reached. Consequently, UAVs have also been used in many ecological settings. These include invasive species mapping (Hill et al., 2017; Müllerová et al., 2017; de S et al., 2018; Martin et al., 2018; Kattenborn et al., 2019), wildlife assessment (Andrew and Shephard, 2017; Rey et al., 2017; Hollings et al., 2018; Christiansen et al., 2019; Eikelboom et al., 2019), and plant biodiversity estimation (Getzin et al., 2012), including object-based species classification (Lu and He, 2017). Moreover, UAVs have been used to track spatial patterns in phenology (Neumann et al., 2020) and flowering of invasive species (de S et al., 2018).

Remote flower mapping in a grassland containing many species is a challenging task because the structures are fine and flowers might be occluded by other plants. Current approaches of automated flower mapping work with image resolutions in the range of centimeters or even meters per pixel (Abdel-Rahman et al., 2015; Landmann et al., 2015; Chen et al., 2019) and are therefore not suited to detect individual flowers and differentiate between flower species of similar color. Other approaches are tailored to a single species (Horton et al., 2017; Campbell and Fearns, 2018) and are not applicable to a wide range of use cases.

Recently, deep-learning-based classification methods that are able to utilize the details of ultra-high-resolution image data have been developed. In particular, deep convolutional neural networks (CNNs) have revolutionized image interpretation by improving the accuracy of object detection and classification tasks. A deep CNN is a network with many layers. It takes the pixels of an image as input and, as output, predicts the likelihood for each class label it has been trained on. Internally, it applies thousands of learned filters to all regions of the image and in the end combines them to find the likelihood for each class label. The end-to-end approach of deep learning methods allows automatic detection of important features without human interaction because the networks automatically learn which features are the most important ones. Recently, such approaches have been introduced to detect and count animals (Rey et al., 2017; Eikelboom et al., 2019) and plants (Eikelboom et al., 2019; Kattenborn et al., 2019; Osco et al., 2020) in an ecological context.

In this article, we present a deep-learning-based method to collect information about flower abundance and distribution in grasslands from drone-based aerial images. To evaluate its performance, we addressed several questions:

1. How does manual counting of flowers compare with tablet-assisted annotations on high-resolution aerial imagery?
2. How does drone-based, automated deep learning flower counting compare with a manual assessment?
3. How does a drone-based, automated flower mapping of a whole meadow compare with extrapolation from the counting of flowers in distinct sample squares?

## 2. Materials and Methods

### 2.1. Overview

**Figure 1:**
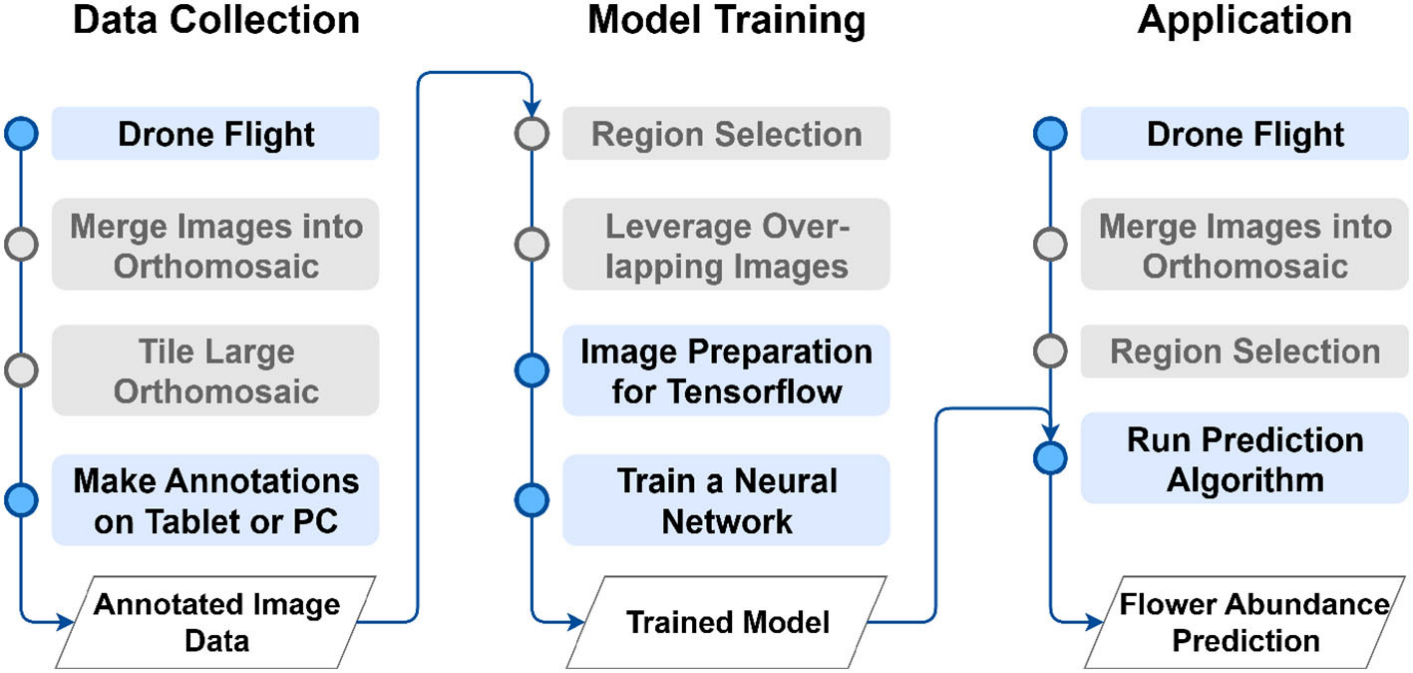
Overview of the proposed method. Gray-colored steps might not be necessary for some use cases. For a comprehensive explanation of the main phases data collection (Section 2.2), model training (Section 2.3) and application (Section 2.4) please refer to the corresponding section.

The proposed method can be divided into the three main phases of data collection (Section 2.2), the model training (Section 2.3), and the application to unseen images (Section 2.4) as depicted in Figure 1.

### 2.2. Data Collection

#### 2.2.1. Dataset

The dataset on which the method was evaluated consisted of 10,000 annotated flowers. The aerial images were captured at two sites and on 5 days from a flight height of 19 m and a ground sampling distance of approximately 1.5 mm per pixel. For the collection of the flower dataset, a drone model called Transformer UAV (Copting GmbH, 2017) and a DJI Matrice 600 PRO (SZ DJI Technology Co., Ltd., 2018) were used. Both drones were programmed to fly along a predefined route such that the area was fully covered and the images had an overlap of 90%. Attached to the drone was a Sony ILCE-7RM2 (Sony Corporation, 2015) camera that took 42.2-megapixel photos in combination with a Zeiss Batis 1.8/85 telephoto lens (Carl Zeiss AG, 2017). The weather was sunny on all flight days. One of the two sites has been managed extensively during the last 15 years such that the plant diversity in this meadow was very high. Forty flowering plant species were found between May 23rd and July 3rd of 2019. Approximately half of these species were omitted in the analysis because too few samples (less than 50) were present in the survey plots. As summarized in Table 1, some flowers were combined into groups because they had few annotated samples or they looked similar to other flowers. Because the individual flowers within an inflorescence could rarely be identified in the drone-based images, all inflorescences were annotated as one flower instance. Subsequently, when we refer to the term flower, inflorescences were included as well.

**Table 1:** Groups of plant species that were combined into one group (table header).

***Ranunculus* species (*n* = 474)**	***Lotus corniculatus* (3,271)**	***Galium mollugo* (659)**	***Crepis biennis* (159)**	***Centaurea jacea* (805)**
-*Ranunculus bulbosus* (442)	-*Lotus corniculatus* (2926)	-*Galium mollugo* (202)	-*Crepis biennis* (89)	-*Centaurea jacea* (786)
-*Ranunculus friesianus* (8)	-*Lathyrus pratensis* (345)	-*Achillea millefolium* (338)	-*Leontodon hispidus* (10)	-*Lychnis flos-cuculi* (19)
-*Ranunculus acris* (24)		-*Daucus carota* (65)	-*Tragopogon pratensis* (8)	
		-*Carum carvi* (54)	-*Picris hieracioides* (52)	

#### 2.2.2. Traditional Data Acquisition

Traditionally, the most commonly applied method to obtain information about flower abundance in the flowering vegetation of a focal land use area is a direct visual assessment of the flowering vegetation by an observer in the field; the observer counts or estimates the flowers of each flowering plant “by hand” (i.e., manually) within survey plots of appropriate numbers and sizes for the specific study, which are distributed within the area of interest (Albrecht et al., 2007; Szigeti et al., 2016; Bartual et al., 2019). In our study, flower types ranged from simple (e.g., Violaceae) to more complex types in which flowers are arranged in clusters of various sizes and shapes (e.g., Apiaceae, Asteraceae). These inflorescences were classified according to Pywell et al. (2004) and Bartual et al. (2019) and were counted for each flowering dicotyledon plant species in each survey plot. Once the flowers present within the survey plots were counted or estimated, these numbers were extrapolated to the size of the whole area of interest by multiplying the counts by a factor corresponding to the relative size of the plots to the total area of the field. When applied with adequate numbers and sizes of plots, this method has been shown to provide reliable estimates of the abundance of flowers in an area of interest (Szigeti et al., 2016). In the present study, we randomly located 15 survey plots (1 m by 1 m) in the study grassland. This large number of plots was used to account for the typically high heterogeneity in the composition of the flowering plant communities and their spatial distribution in grasslands (Bartual et al., 2019). We carried out this traditional approach of manual counting in parallel to each iteration of the drone-based data acquisition method and used it as a baseline.

#### 2.2.3. Drone-Based Data Acquisition

Because one aim of this study was to carry out a multi-temporal analysis of the flower abundance, we placed ground control points (GCPs) within the test region to geographically align the results of subsequent flights. The placement of the GCPs was simulated with the PhenoFly flight planning tool described by Roth et al. (2018b) to get an intuition on how to distribute the GCPs. The GCPs were then distributed across the meadow with a squared layout with the distance between the GCPs ranging between 4 and 7 m. With this setup, one to two GCPs were visible in each image. Each GCP had a size of 0.15 m, which corresponds to approximately 150 pixels. The exact coordinates of all these GCPs were collected with a Differential Global Navigation Satellite System (R10, Trimble Ltd., Sunnyvale, CA) with swipos-GIS/GEO RTK (real-time kinematic) correction (Federal Office of Topography Swisstopo, Wabern, Switzerland), resulting in a horizontal accuracy of 0.008 m and a vertical accuracy of 0.015 m. Later, they were used in the software Agisoft (Agisoft, 2019) as described below. Having the GCPs in place, the drone was flown along a predefined route across the field.

After the flight, the relative positions and orientation of the aerial images were reconstructed and merged together into a large orthomosaic. An orthomosaic is a visual representation of an area, created from many photos that were stitched together in a geometrically corrected way. We used the Structure from Motion approach (Ullman, 1979; Harwin and Lucieer, 2012) implemented in the software Agisoft Metashape Version 1.5.3 (Agisoft, 2019). Agisoft takes all aerial images as input and aligns them *via* bundle adjustment. This procedure allows generating a point cloud of the topography of the surveyed area. From the point cloud, a digital surface model was generated to orthorectify the orthomosaic. During orthomosaic generation, we used the option *blending disabled* to prohibit smearing of the original information of the images in the orthomosaic. The orthomosaic was georeferenced based on the GCP position.

Agisoft automatically detects the unique pattern on the GCPs to map the GPS coordinates to each of them. The advantage of providing the positions of the GCPs in the field is that the resulting orthomosaic is georeferenced. The georeferenced orthomosaic was later used to display the user's position in the Android application FieldAnnotator and to be able to copy annotations to the single orthorectified images that were georeferenced (refer to Sections 2.2.4 and 2.2.5 for further information).

#### 2.2.4. Annotation

**Figure 2:**
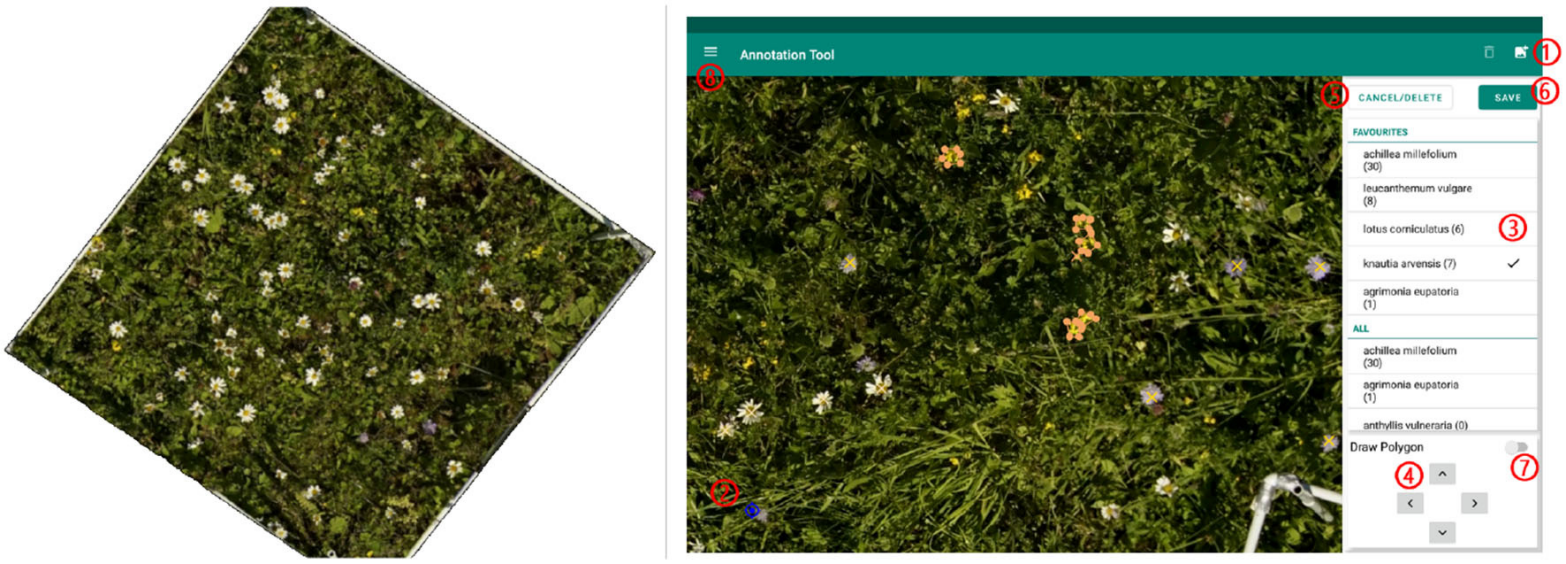
(Left) Example of area extracted from a sample square to be annotated. (Right) Screenshot of the FieldAnnotator during the annotation process. The numbers highlight the controles of the FieldAnnotator. For a detailed description please refer to Supplementary Section A.1.

On the georeferenced orthomosaic, the areas of all sample squares were extracted and all flowers annotated. For annotating, we used the program LabelMe (Wada, 2016) and an Android tablet application called PhenoAnnotator (Figure 2) that we specifically developed for this purpose (for a detailed description, refer to Supplementary Section A.1). The FieldAnnotator can be found at https://github.com/tschutli/Phenotator-Toolbox or at the Google Play Store. Android tablets were not capable of handling large orthomosaics (around 50, 000 times 50, 000 pixels for an area of 30 m by 30 m). Therefore, the orthomosaics were tiled into small chunks of 256 times 256 pixels at various zoom levels before these tiles were then imported into the FieldAnnotator application. The resulting annotations were stored in a json file.

#### 2.2.5. Leveraging Overlapping Images

Because the camera attached to the drone captures a large number of highly overlapping images, the overlapping images can be used to create additional training data with flowers pictured from a slightly different angle on each image. We transferred the geolocation of each flower mapped on one image to the other images. Because grasslands have a very complex structure, some of the copied annotations were slightly shifted within the overlapping images. To correct for the shift, an additional script was written to let the user view and adjust all annotations in the LabelMe application. These slight adjustments of the annotations took significantly less time than annotating the data from scratch.

### 2.3. Model Training

#### 2.3.1. Selecting Regions of Interest in Annotated Images

In case the images were only partly annotated, we developed a script that allows the user to cut out certain regions (polygon shaped) from the images. Only the image pixels within these selected regions were kept while the rest of the image pixels were over-written with black. This procedure ensured that the TensorFlow model (Abadi et al., 2015) did not learn to classify non-annotated flowers as the background class.

#### 2.3.2. Image Preparation for TensorFlow

As a result of these preparations, the training data consisted of image files alongside with json files containing the annotations. To prepare the data to be imported into TensorFlow, we first split up the images into tiles. The default tile size was set to 450 times 450 pixels. These image tiles were then upscaled by a factor of two to tiles of 900 times 900 pixels as suggested by Hu and Ramanan (2017) and as justified in the Supplementary Section A.2. The tiles were overlapping such that flowers positioned on the edge of two tiles were not lost as training data but were always present as a whole in at least one tile. Additionally, all annotations (including point and polygon annotations) were converted to bounding boxes. Finally, the images were split up into training, test and validation sets.

#### 2.3.3. Neural Network Training

The core of the pipeline consisted of a CNN. We used the Faster R-CNN architecture (Ren et al., 2015). This architecture outputs the bounding box coordinates of the objects it recognizes on an input image. The Faster R-CNN architecture requires more computing power than other architectures do, but it has been shown to perform well on aerial and other high-resolution images (Carlet and Abayowa, 2017; Huang et al., 2017). Because the default configuration of the Faster R-CNN architecture is not optimized to detect very small objects (Huang et al., 2017; Zhang et al., 2017) of only a few pixels in diameter (as it is the case for flowers in aerial images), we adjusted some parameters (refer to Supplementary Section A.2 for experiment results on different parameter combinations). Additionally, we used some typical data augmentation techniques to increase the diversity of our dataset, namely, random horizontal and vertical flips, random brightness adjustments, random contrast adjustments, random saturation adjustments, and random box jittering^1^.

During training, the validation set was used to decide when to change the learning rate and when to stop training. Every 2,500 steps, the training was paused and the prediction algorithm followed by the evaluation algorithm was run on the validation set. The learning rate was adjusted if for the last 15,000 steps no further improvements were made. After adjusting the learning rate two times, from 3E-04 to 3E-05 and from 3E-05 to 3E-06, the training was stopped if for 15,000 steps no improvement in the performance was seen. The number of 15,000 steps was chosen empirically based on an evaluation of initial results that showed that no model was further improved after it did not improve for 15,000 steps. Reducing the learning rate two times by a factor of 10 was adapted from the Faster R-CNN default configuration. The evaluation metric could be chosen as either the F1 score or the mean average precision (mAP). Section 2.4 further explains the prediction and evaluation processes.

The number of training examples can vary greatly from class to class. Therefore, each class was assigned a weight. The weight was inversely proportional to the number of training examples and influenced the loss function during training. This weighting ensured that the network did not just optimize to detect the most common classes. As a consequence, each mistake in a less common class had a much higher penalty to the loss function. Once a network was fully trained, it was exported as an inference graph. This exported inference graph could then be used by the prediction and evaluation scripts described in Section 2.4.

### 2.4. Application to Unseen Images

#### 2.4.1. Predictions

The trained network can be used to make predictions on images of arbitrary size (e.g., orthomosaics) provided they have a ground sampling distance similar to that of the training images. The pipeline handles the tiling of large images as well as the reassembling of the prediction results from the single tiles. Optionally, a region of interest can be selected within an image. As a consequence, only the flower abundance within this region of interest is assessed by the prediction algorithm.

The prediction algorithm draws the bounding boxes of all detected flowers onto the image and saves the statistics about the flower abundance to a json file. To improve the prediction accuracy, the tiles had an overlap of 100 pixels by default. This overlap ensured that as long as a flower was not larger than 100 pixels in diameter, it was fully visible on at least one tile. Error-prone predictions close to or on the edge of a tile could therefore be ignored because they were fully covered on the adjacent tile. Nevertheless, having this overlap introduced the problem of duplicate predictions. This problem was mitigated by applying non-maximum suppression with an intersection-over-union (iou) threshold of 0.3, similar to the threshold applied by Ozge Unel et al. (2019), such that for all predictions that had an overlap of more than 30%, only the one with the highest confidence score was kept.

#### 2.4.2. Evaluations

To evaluate the performance of a model, the predictions on the test set were compared with the validation annotations of the test set. The main metrics of interest were precision and recall. To compute precision and recall values, the true positive (TP), false positive (FP), and false negative (FN) predictions had to be known. To obtain these values, the predictions were sorted by their confidence. Then, all predictions were compared with ground truth bounding boxes of the same label. To compare two bounding boxes, the *iou* formula was used:


$$iou=\frac{intersection\;area}{area\;of\;union}$$

If the highest *iou* value was greater than a given threshold value (default of 0.3), the corresponding ground truth box was marked as used and the prediction was marked as TP. If the highest *iou* value was less than the threshold value, the prediction was marked as FP. After this process was done for each prediction, all ground truth entries that were not marked as used were counted as FN. Having the TP, FP and FN numbers, the precision and recall values were calculated using the following formulas:


$$\begin{array}{l} precision=\frac{TP}{TP+FP}\\ recall=\frac{TP}{TP+FN} \end{array}$$

Additionally, we calculated the F1 score as follows:


$$F1=2\cdot \frac{precision\cdot recall}{precision+recall}$$

The better the precision and recall values, the better is the F1 score. It rates precision and recall equally and reaches its maximum of one at perfect precision and recall. As an alternative to the F1 score, the mAP as defined in the PASCAL Visual Object Classes Challenge Development Kit (Everingham and Winn, 2011) was used to rate a model's performance.

#### 2.4.3. Visualizations

The pipeline offers various options for visualizing the results. Apart from drawing the predictions as colored bounding boxes onto the images, erroneous predictions can be highlighted. Additionally, heatmaps that visualize the density distribution of the flowers can be generated from the prediction output. The size of the kernel for the flower density mapping is customizable. Optionally, the heatmap can be drawn directly onto the image. The heatmaps can be generated for an individual class or for all classes. If the input images are georeferenced, there is the option to generate one heatmap from a collection of images. If the images are overlapping, the heatmap indicates the average number of flowers found at a particular position. Furthermore, the user can provide the geocoordinates of the upper left and lower right corner of the desired output region. The script will then output a heatmap of exactly that region. This option allows for time series generations. Example results of such time series generations can be viewed in Section 3.4.

### 2.5. Impact of Ground Sampling Distance

To investigate the impact of different ground sampling distances, the training, test, and validation images were first scaled down to the desired ground resolution and then scaled up again to their original resolution. After upscaling, all datasets had the same ground sampling distance as the original images. This procedure ensured that the flowers' sizes (in image pixels) were large enough to be detectable by the Faster R-CNN network architecture and prevented performance losses caused by this problem as described by Hu and Ramanan (2017). For each ground resolution, a network was trained and evaluated with the processed training images.

## 3. Results

Comparing human counting with drone-based automated mapping has three aspects. First, we assessed the differences between the manual counting and the tablet annotations within patches of vegetation marked with wooden squares (vegetation squares). Second, we evaluated the performance of the deep-learning-based flower detection algorithm on the images within the vegetation squares. Third, we compared the automated estimates for the whole meadow with the extrapolation from the manual counts within the vegetation squares to the whole area of the meadow.

### 3.1. Manual Counting vs. Drone-Based Image Tablet Annotations

**Figure 3:**
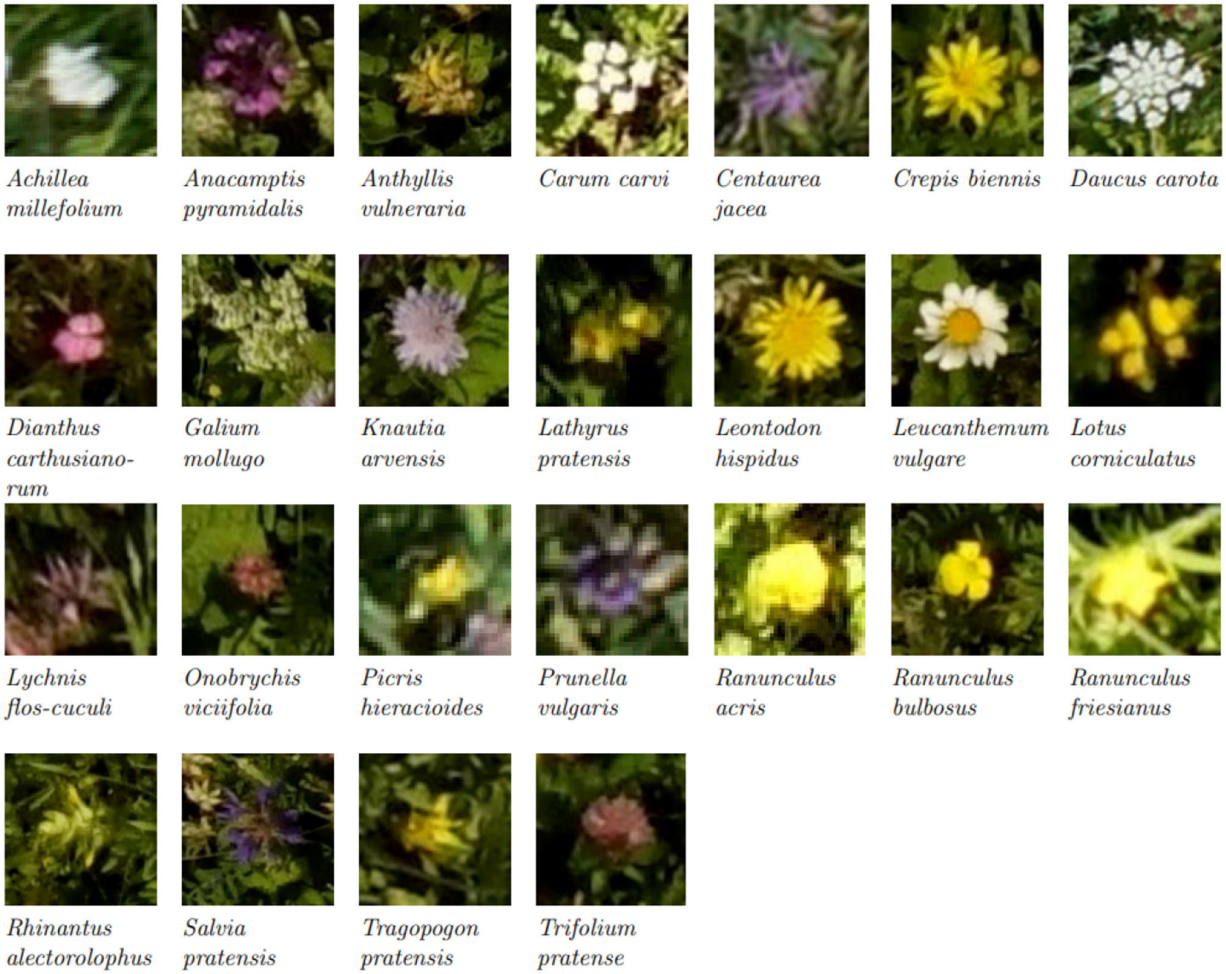
Excerpts from aerial images of the most common flower species. Please note that the images have been scaled to show the flower heads, and thus the pixel size is not consistent between the excerpts. (For images with real relative scaling, please refer to Supplementary Section A.3).

We compared the flower heads annotated within the vegetation squares on the drone-based aerial images *via* the tablet application with those manually counted by an observer. Table 2 lists the results for a representative subset of all flowers found within the test fields. For *Salvia pratensis, Ranunculus* species, and *Centaurea jacea*, the tablet and manual counts aligned well. For *Leucanthemum vulgare* and *Knautia arvensis*, more flowers were annotated on the tablet. For the other four species, fewer flowers were annotated on the tablet. For *Medicago lupulina*, only very few instances were annotated on the tablet. Refer to Figure 3 for visualizations of 25 flower species found within the test fields.

**Table 2:** Comparison of selected manually counted total numbers with tablet annotations.

**Flower**	**Manually counted**	**Tablet annotations**	**Ratio**
*Leucanthemum vulgare*	724	960	1.3
*Onobrychis viciifolia*	483	105	0.2
*Lotus corniculatus*	1,943	748	0.4
*Salvia pratensis*	142	127	0.9
*Ranunculus* species	431	474	1.1
*Knautia arvensis*	371	471	1.3
*Trifolium pratense*	129	72	0.6
*Medicago lupulina*	117	5	0.0
*Centaurea jacea*	25	28	1.1

*The last column shows the ratio of the tablet annotations divided by the manually counted flowers*.

### 3.2. Algorithm Performance Inside Survey Plots

We compared the tablet annotations with the deep learning predictions within the survey plots. The prediction performance for each flower species can be obtained from **Table 4**. A prediction was considered for the comparison if its confidence score was greater than 0.2. The overall precision and recall were 87.0 and 84.2%, respectively. The vast majority of the flowers present in the test data of June 14th were *Knautia arvensis, Leucanthemum vulgare*, and *Lotus corniculatus*. These three flower species performed well, and, therefore, the good overall score was mainly determined by these three flower species. All the other flower species performed worse than the overall performance indicates.

Table 3 shows the confusion matrix of this experiment. It was striking that there were only a few confusions between different flower species. The much more common cases of confusion were that flowers were predicted where there was none and flowers were not predicted where they should be. The green entries denote the correctly predicted flowers.

**Table 3:** The table shows the confusion matrix.

	***A. vulneraria***	***C. jacea***	***C. biennis***	***D. carthusianorum***	***G. mollugo***	***K. arvensis***	***L. vulgare***	***L. corniculatus***	***O. viciifolia***	***P. vulgaris***	***Ranunculus* species**	***R. alectorolophus***	***S. pratensis***	***T. pratense***	**False Negatives**
*A. vulneraria*	1	-	-	-	-	-	-	3	-	-	-	-	-	-	2
*C. jacea*	-	27	-	-	-	17	-	-	-	1	-	-	3	3	2
*C. biennis*	-	-	14	-	-	-	-	5	-	-	-	-	-	-	2
*D. carthusianorum*	-	3	-	8	-	1	-	-	10	-	-	-	-	6	6
*G. mollugo*	-	-	-	-	8	-	-	-	-	-	-	-	-	-	8
*K. arvensis*	-	-	-	-	-	412	1	-	-	-	-	-	2	-	23
*L. vulgare*	-	-	1	-	1	4	906	-	-	-	-	-	1	-	109
*L. corniculatus*	-	-	6	-	-	-	1	877	-	-	-	-	-	-	142
*O. viciifolia*	-	1	-	-	-	1	-	-	45	-	-	-	-	11	37
*P. vulgaris*	-	-	-	-	-	-	-	-	-	-	-	-	-	-	-
*Ranunculus* species	-	-	-	-	-	-	-	-	-	-	-	-	-	-	-
*R. alectorolophus*	-	-	-	-	-	-	-	1	-	-	-	8	-	-	17
*S. pratensis*	-	-	-	-	-	-	-	-	-	-	-	-	12	-	3
*T. pratense*	-	-	-	-	-	1	-	-	-	-	-	-	-	4	2
False positives	4	6	17	-	32	24	31	117	3	-	1	5	6	18	-

*The columns represent what the model predicted, and the rows represent what the model should have predicted (the ground truth). The green, red, orange, and brown numbers denote TP, FP, FN, and confusions between two flower species, respectively*.

Table 4 shows that the flowers with little training data tended to not perform well. The question is whether this low performance was due to the lack of enough training data or because assigning an inversely proportional weight to each class during training is not sufficient to regularize the loss function. Therefore, we trained a separate network in which the three best performing flowers (*Leucanthemum vulgare, Lotus corniculatus* and *Knautia arvensis*) were ignored and treated as background. With the mAP rising from 25.2 to 31.5% (F1 score improving from 47.0 to 51.3%), a certain improvement could be seen. Therefore, the possibility of leveraging two separately trained networks was not further evaluated.

**Table 4:** Performance of the prediction algorithm on all flower species present in the field on June 14th.

**Flower species**	**Training instances**	**Test instances**	**Precision (%)**	**Recall (%)**	**mAP**	**F1 Score**
*A. vulneraria*	196	6	20.0	16.7	0.056	0.182
*C. jacea*	742	53	73.0	50.9	0.382	0.600
*C. biennis*	124	21	36.8	66.7	0.325	0.475
*D. carthusianorum*	20	34	100.0	23.5	0.235	0.381
*G. mollugo*	546	16	19.5	50.0	0.100	0.281
*K. arvensis*	429	438	89.6	94.1	0.879	0.918
*L. vulgare*	928	1,022	96.5	88.6	0.861	0.924
*L. corniculatus*	2,153	1,026	87.4	85.5	0.772	0.864
*O. viciifolia*	92	95	77.6	47.4	0.407	0.588
*R. alectorolophus*	23	26	61.5	30.8	0.218	0.410
*S. pratensis*	133	15	50.0	80.0	0.436	0.615
*T. pratense*	109	7	9.5	57.1	0.104	0.163
Overall	5495	2759	87.0	84.2	0.398	0.855

*The numbers in the Training Instances and Test Instances columns refer to the ground truth annotations. The overall scores of the performance metrics were weighted means. mAP = mean average precision*.

**Figure 4:**
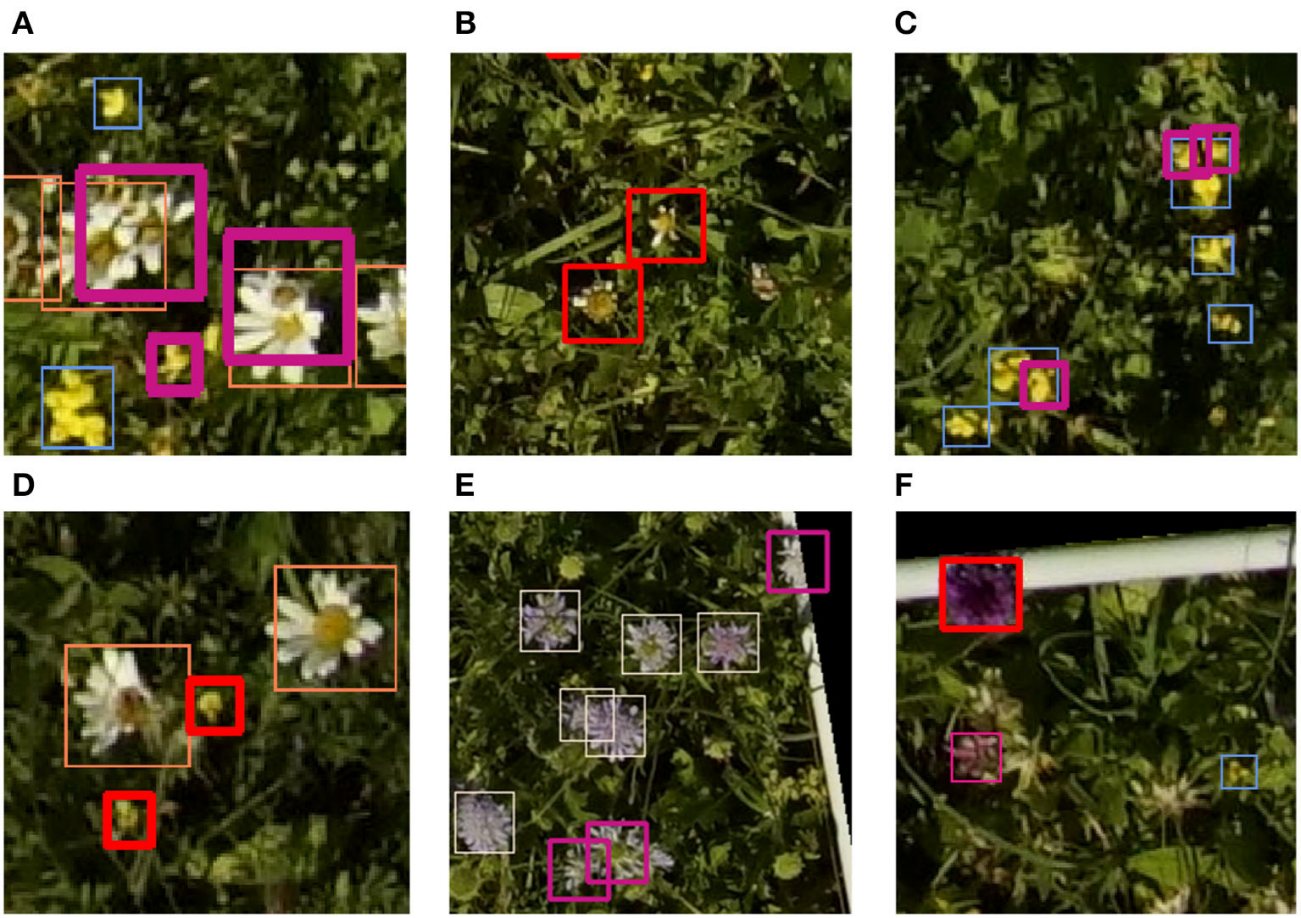
Selection of typical mispredictions. All thin bounding boxes are correct predictions. The bold red bounding boxes denote false positive and the bold violet bounding boxes denote false negative predictions. There are various explanations for the mispredictions: Overlapping flowers **(A)**, partially withered flowers **(B,E)**, collections of flowers **(C)**, missing ground truth annotations **(D)** and flowers that are missing in the training data **(F)**.

When looking at the predictions, there were various sources of errors apparent. Some examples can be seen in Figure 4. For *Leucanthemum vulgare*, a typical error occurred where two instances were very close to each other as in image a). In that case, often only one of the two flowers was detected. The missing annotation was not caused by the non-maximum suppression algorithm, as a closer look disclosed. Another typical source of errors was flowers that were on the verge of fading. In the case of image b), two flowers were detected that were not annotated in the ground truth because the botanical expert considered the flowers to be faded already. Even when manually counting the flowers, it was sometimes difficult to decide if a flower should be counted or not because of the seamless transition from blooming to faded. Two main problems existed for *Lotus corniculatus*. First, the blooms of *Lotus corniculatus* were often arranged as small inflorescences, as visible in the image a) to the bottom left or in image c). In some cases, the network predicted the blooms of an inflorescence as individual instances whereas in the ground truth, the whole inflorescence was annotated as one instance. The opposite case was common as well. The second problem of *Lotus corniculatus* was FP predictions caused by missing ground truth annotations [as in image d)]. These problems are further discussed in Section 4.1. The main error source for *Knautia arvensis* were blooms that looked different because they were wilting as for example in image e). In image f), the model erroneously predicted a *Knautia arvensis* where there was an *Anacamptis pyramidalis* flower. *Anacamptis pyramidalis* was not included in the training because too few training instances existed.

### 3.3. Prediction on a Whole Meadow

Counting flowers in small, representative areas of a field and extrapolating these counts to the area of the whole field is a common method in field ecology. We compared the predictions of the deep learning model on the whole test field with the extrapolation of the flowers manually counted within the vegetation squares. The numbers of manually counted flowers were extrapolated to the size of the whole field, which was 730 m^2^. Table 5 lists all flowers that were detected reasonably well inside the survey plots by the deep learning model. For each flower species, the number of deep learning detections in the whole field was listed as well as the number of flowers predicted by the extrapolation of the manual counts.

**Table 5:** Predictions on the whole field of 730 square meters.

**Flower**	**Drone-based prediction**	**Extrapolation of manual counts**	**Relative difference (%)**
*Centaurea jacea*	456	505	10.7
*Knautia arvensis*	8,059	8,308	3.1
*Leucanthemum vulgare*	7,044	10,778	53.0
Lotus corniculatus^*^	50,365	51,139	1.5
*Onobrychis viciifolia*	595	3,761	532
*Salvia pratensis*	209	673	222

* *The 50, 365 predicted Lotus corniculatus flowers were calculated as the multiplicative of the actual predictions of the network (19389) and a ratio of 2.6. The numbers in Table 2 suggest that there are on average 2.6 blooms per prediction*.

For *Centaurea jacea, Knautia arvensis*, and *Lotus corniculatus*, the number of drone-based predictions was very similar to the extrapolation of the manually counted number of flowers. The results were within 11, 3, and 2%, respectively. According to heatmaps generated from the drone-based predictions (see Section 3.4), these were also the flowers that were relatively evenly distributed. The extrapolation of the manually counted number of *Leucanthemum vulgare* flowers was 53% higher than the number of drone-based predictions.

### 3.4. Density Distribution Maps

**Figure 5:**
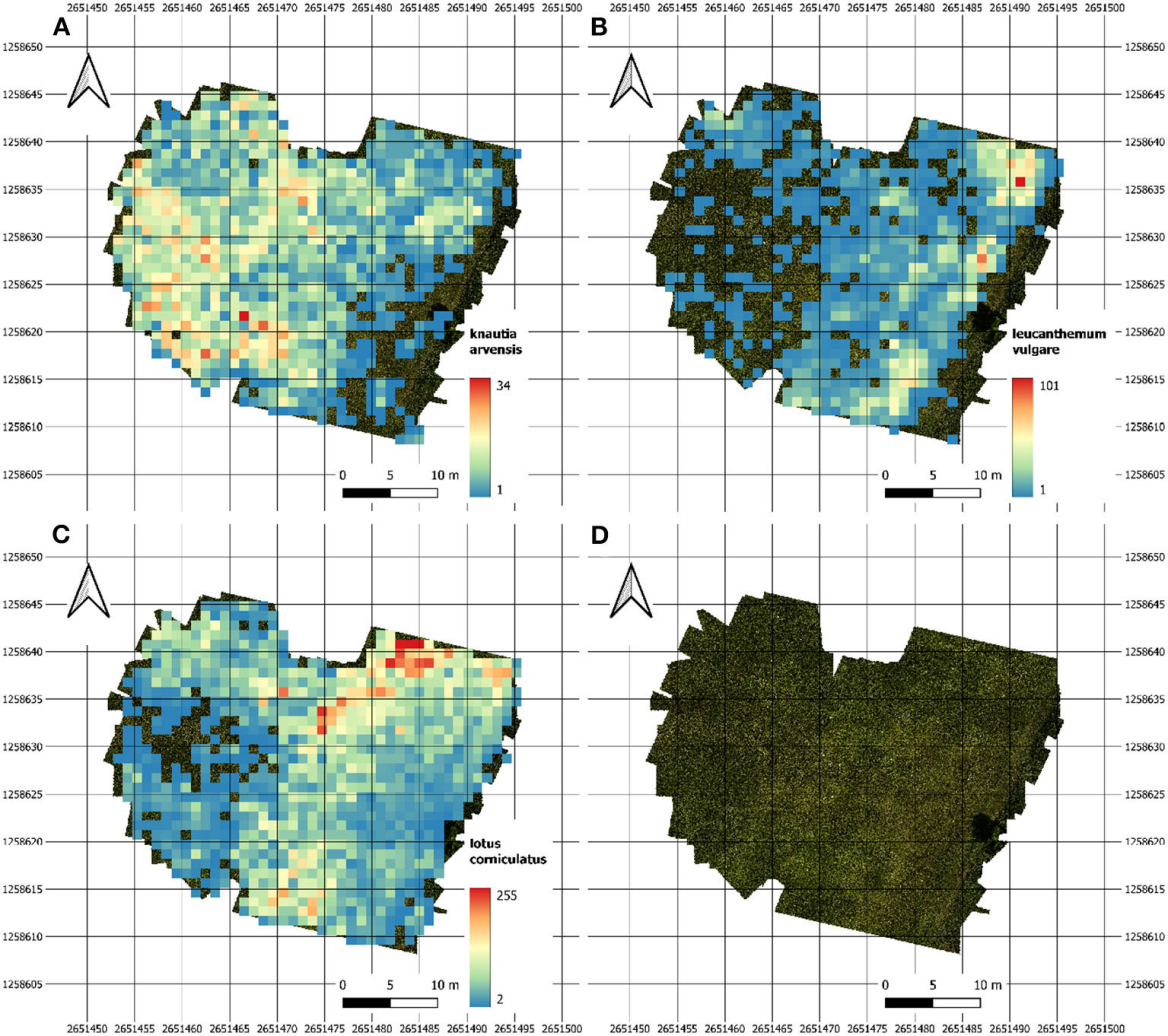
Heatmaps of our main test site showing the abundance density for **(A)** *Knautia arvensis*, **(B)** *Leucanthemum vulgare*, and **(C)** *Lotus corniculatus*. Image **(D)** depicts the image coverage of the field.

The heatmaps in Figure 5 depict the abundance of selected individual flowers in one of our test fields on June 14th. The three heatmaps for *Leucanthemum vulgare, Lotus corniculatus*, and *Knautia arvensis* were generated from the orthomosaic.

**Figure 6:**
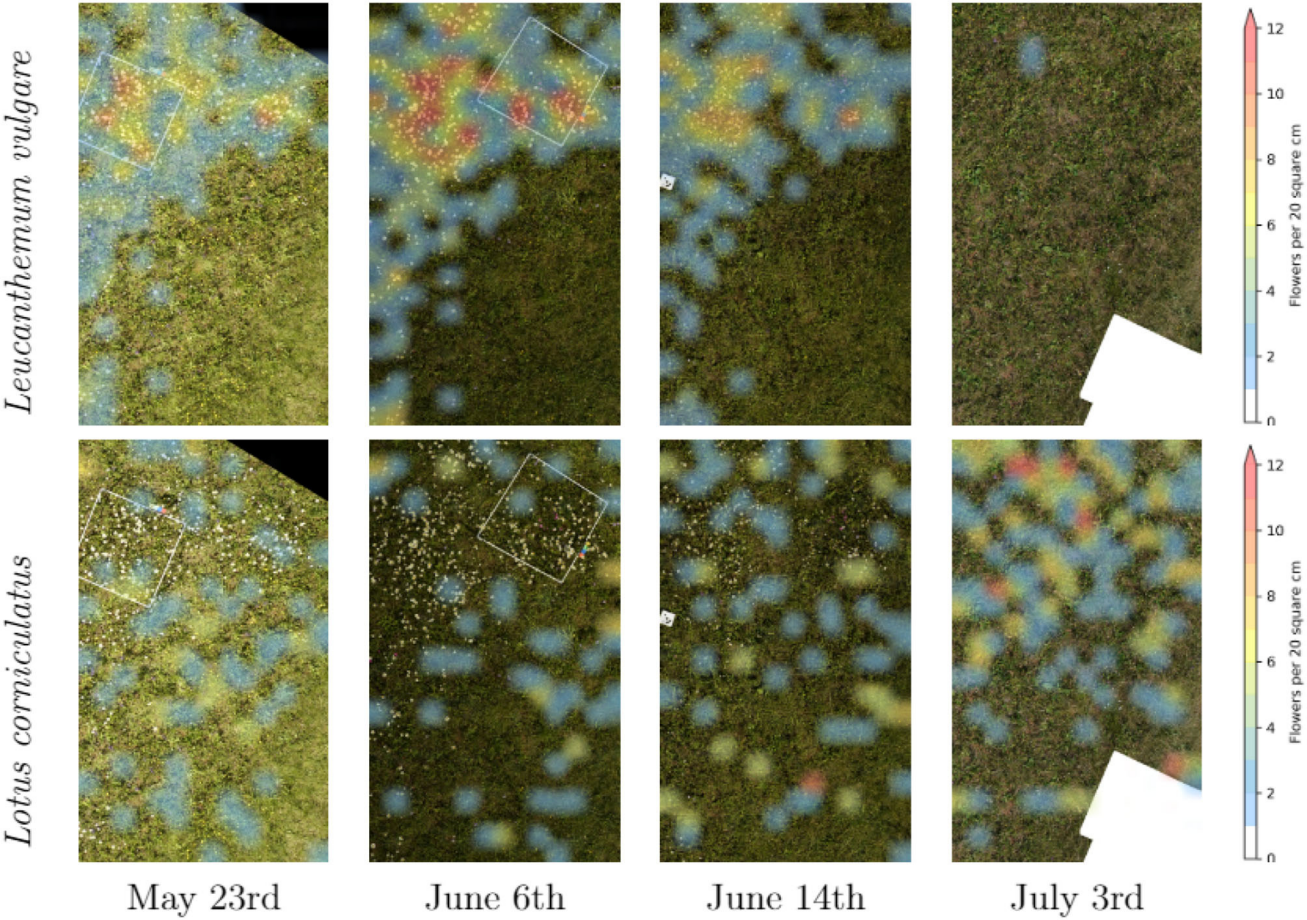
Time series of the distribution of *Leucanthemum vulgare* and *Lotus corniculatus* in our main test field.

Figure 6 contains a time series of an excerpt of our main test site. It illustrates the difference in the abundance evolution of *Leucanthemum vulgare* and *Lotus corniculatus*. It is conspicuous that the *Lotus corniculatus* population was much more evenly distributed than the *Leucanthemum vulgare* population. *Leucanthemum vulgare* had a peak population on June 6th, whereas on July 3rd, the population was almost completely faded. The peak population of *Lotus corniculatus* was much less pronounced.

### 3.5. Impact of Ground Sampling Distance

**Figure 7:**
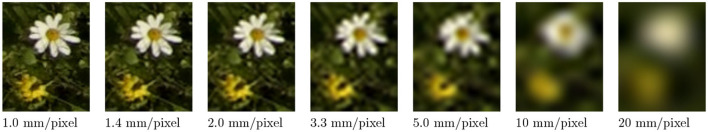
Ground sampling distance degradation on an excerpt of an aerial image.

**Figure 8:**
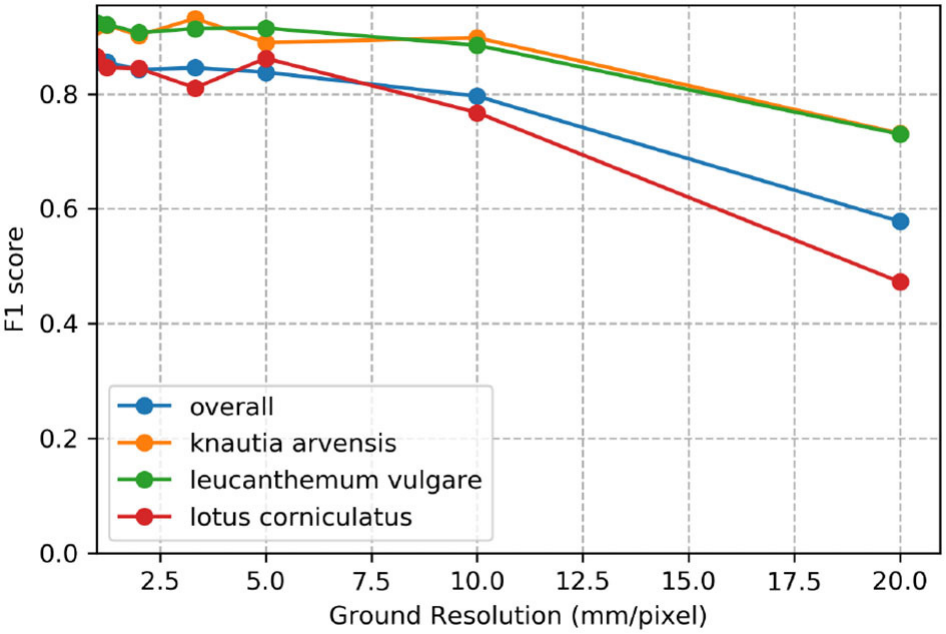
Evolution of the F1 score over various simulated ground resolutions.

**Figure 9:**
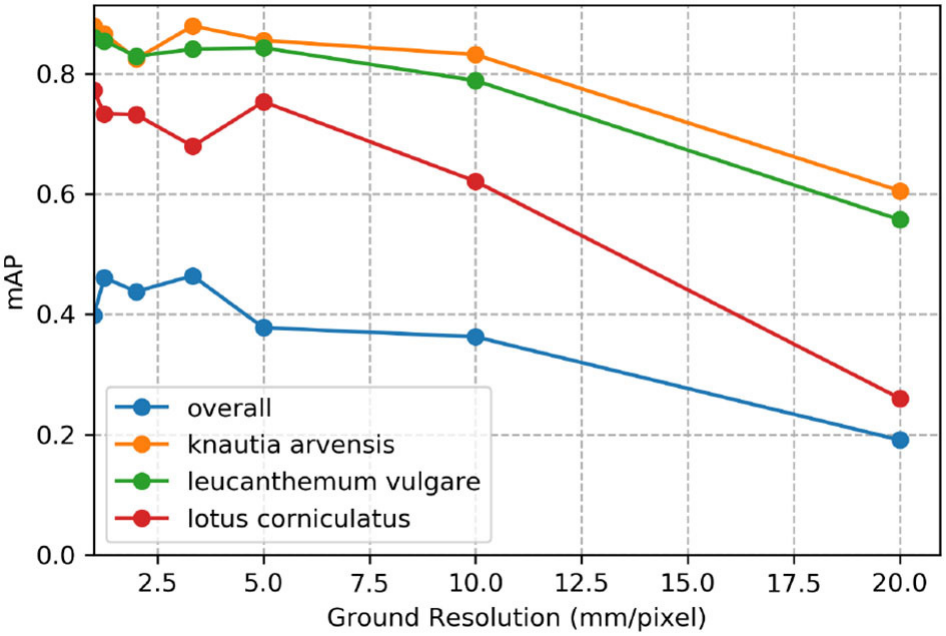
Evolution of the mean average precision (mAP) over various simulated ground resolutions.

Figure 7 demonstrates the effect of decreasing ground sampling distance on an exemplary excerpt of an aerial image containing a *Leucanthemum vulgare* flower and a *Lotus corniculatus* inflorescence. Figures 8, 9 illustrate the effect of decreasing ground resolution on the F1 score and the mAP, respectively. Both figures show that down to a ground sampling distance (GSD) of 5 mm, there was a marginal decrease in prediction performance. Further decreasing the GSD to 10 and 20 mm per pixel had noticeable negative effects on the model's performance. As expected, the performance of small flowers such as those of *Lotus corniculatus* decreased disproportionately because at a certain ground resolution they simply became indistinguishable. The average size of a *Lotus corniculatus* flower was around 16 mm. The performance of larger flowers such as those of *Leucanthemum vulgare* (40 mm) and *Knautia arvensis* (34 mm) degraded notably more slowly. The graphs for the precision and recall metrics were omitted because the trends were equivalent to the trends of the F1 score and the mAP metric.

## 4. Discussion

### 4.1. Tablet-Assisted Annotations in Vegetation Squares

We evaluated different approaches to map flowers in grasslands. We used manual counting within survey plots as a baseline and compared it with tablet annotations on drone-based aerial images of the survey plots and automated deep-learning-based mapping within the survey plots. The advantage of being able to annotate the images on a tablet is that some flowers can be very hard to distinguish on the images. If one can compare the image with the actual flowers on site, the quality of the training data can be improved, and the number of false annotations is thus minimized.

Section 3.1 shows that some flowers had more tablet annotations on the images than were manually counted by an observer within the survey plots. These were flowers of *Leucanthenum vulgare, Ranunculus* species, *Knautia arvensis*, and *Centaurea jacea*. An explanation for this finding is that manually counting flowers requires a high level of concentration. Mistakes happen very easily when many flowers are present within a small area. Annotating on an image has the advantage that flowers are marked and therefore the risk of counting a flower twice or overlooking a flower is minimized. On the other hand, some flowers were hardly visible on the drone-based images, and therefore significantly fewer instances were counted in the tablet annotations compared with the manually counted data. *Onobrychis viciifolia, Medicago lupulina* and to some extent *Trifolium pratense* fall in this category. The flowers of *Medicago lupulina* were too small to be reliably identifiable on the drone-based images. Those of *Trifolium pratense* and *Onobrychis viciifolia* would be large enough but were often hardly distinguishable from the background.

### 4.2. Performance of the Detection Algorithm

Whether it is possible to achieve reliable predictions for a certain flower on drone-based images depends on several factors. First, enough training data on the flower in question needs to be available. Our results suggest that with a few hundred instances, good performance can be achieved. Second, also the morphology of the flower has an impact. Flowers such as those of *Galium mollugo* are difficult for an object detection network to predict reliably. The cause seems to be that these flowers can sometimes be very small and, in other cases, multiple instances of the same flower species can cover a large area of partly overlapping inflorescences, making it difficult to separate the single instances. In such cases, it would be interesting to see how an image segmentation network such as U-Net (Ronneberger et al., 2015), which predicts regions (pixels) that belong to a certain class, would perform. Third, the size of a flower should span a certain minimum number of pixels. The good results for *Lotus corniculatus* suggest that a diameter of around 5–10 pixels is sufficient. Besides, these results are likely to be positively influenced by the distinct color and the strong contrast to the background of *Lotus corniculatus* flowers. Other flowers of similar size such as those of *Onobrychis viciifolia* or *Trifolium pratense* performed significantly worse. These flowers were much harder to distinguish from the background. It is evident that distinguishability (mainly driven by contrast) is the fourth main factor that determines the prediction performance of a network for a particular flower.

Generally, it is advised to scale up all images with objects that are smaller than 40 pixels in diameter by a factor of two to improve the performance of a network (Hu and Ramanan, 2017). This is the case for the vast majority of flowers dealt with in this study. The Faster R-CNN architecture is not designed to detect very small objects, such as flowers of just a few pixels in diameter (Huang et al., 2017; Zhang et al., 2017). Therefore, scaling up the images is an appropriate counter measure that helped to improve our results.

When taking a closer look at the results, we found that a substantial portion of mispredictions that negatively influenced the assessed model performance scores (mAP and F1 score) was not fatal. These mispredictions include, for example, FP predictions that were in fact missing annotations in the ground validation data such as the examples in Figure 4. The FP predictions of flowers that were on the verge of fading also fall in this category. The mispredictions caused by the confusion between single flowers and inflorescences of *Lotus corniculatus* as described in Section 3.2 were also not severe.

These mispredictions exemplify the challenges that exist for the training data collection. Even when it is possible to directly compare the image on the tablet with the flowers on site, it is sometimes not clear how to annotate a flower. *Lotus corniculatus* is a good example. Its flowers are often arranged as inflorescences. However, it is not uncommon that there are single flowers that do not belong to the same inflorescence. Because it is often not possible to distinguish the single flowers within an inflorescence, the whole inflorescence is consequently annotated as one flower instance. Unfortunately, there are border cases in which a single flower very close to another inflorescence is annotated as a separate instance in the ground validation but the prediction algorithm includes that flower in the inflorescence and predicts only one bounding box. This situation results in FN predictions for the single flowers very close to each other, as the examples on Figure 4C show. The opposite case that multiple single flowers are predicted separately although they are annotated as an inflorescence with a single bounding box is common as well. The second main problem for *Lotus corniculatus* is that some instances are hardly visible on the images because they are very small. Sometimes they are partly hidden by other vegetation, and occasionally weak motion blur is present and makes it even harder to distinguish between flower and background. This problem also manifests itself in FP and FN predictions. The FP predictions are mainly caused by background areas that look similar to a blurred flower and by real flowers that are not present in the validation annotations (as in Figure 4D). The FN predictions are often flowers that are small and hardly distinguishable. As demonstrated in the example of *Lotus corniculatus* in Table 4, an average number of flowers per annotation can be calculated from the training data and the manually counted data. This value can then be multiplied with the total number of predictions to get the number of flowers.

Data augmentation options are a convenient way of artificially increasing the amount of training data. One should be careful with applying too many augmentation options. Because the flowers do not span a large number of pixels, they are predicted based on minuscule details. Changing these details too much might be counterproductive. Flips and random box jittering can be applied without hesitation. They do not alter the important details but alter only the orientation or the position of the bounding box. Brightness, contrast, and saturation adjustments should be applied moderately. In our experiments, the maximal change rate was a delta of 25%.

### 4.3. Automated Drone-Based Flower Mapping of a Whole Meadow

#### 4.3.1. Comparison With Extrapolations From Vegetation Squares

For some species, we found differences between the extrapolation from the manual counts within the vegetation squares and the drone-based estimations (Table 4). Assuming that the performance of the prediction algorithm on the whole field was similar to the performance within the annotated survey plots, the extrapolation of the manual count data must have been inaccurate. Even when we added 8% to the number of drone-based predictions to compensate for the relatively low recall value of *Leucanthemum vulgare*, the results still had a 47% gap. The extrapolation was based on the manually counted number of flowers, which was less than the number of tablet annotations within the survey plots (as pointed out in Section 3.1). If the tablet-based numbers had been taken, the result of the extrapolation would have been an additional 51% higher, making it in total 131% higher than the drone-based prediction.

The main reason for the bad results for *Onobrychis viciifolia* was that its flowers were very hard to distinguish on the drone-based images. The most probable reason for the unsatisfactory results for *Salvia pratensis* was that the amount of training data was too small to accomplish good results. A likely additional reason could be an unrepresentative choice of survey plot locations for these flowers. When these falsely counted numbers are combined with non-optimally chosen survey plot locations, the extrapolations of the manually counted flowers have the potential to be very inaccurate.

**Figure 10:**
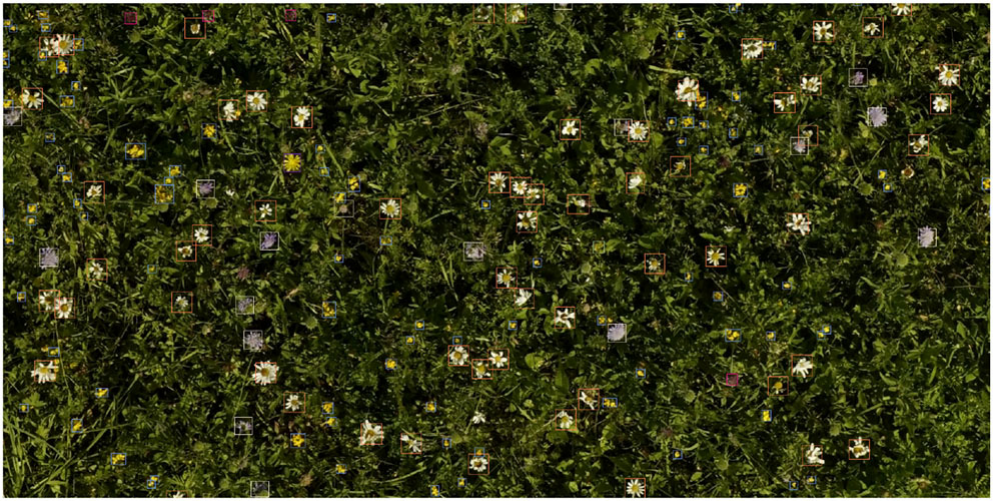
Typical prediction example.

With a reliable flower detection model, the results can be much more accurate than with the extrapolation from the manual counting. Moreover, the drone-based approach has other advantages beyond what can be done with the traditional approach of extrapolating the manually counted numbers of flowers within the survey plots. The combination of deep learning with very-high resolution drone-based remote sensing allows to map objects through space and time (e.g., Figures 5, 6, 10). Moreover, once a trained network is available, manually labeling the species to train the network is no longer necessary. It is sufficient to fly the drone over the meadow and let the deep learning algorithm predict the species. The prediction time of the trained deep learning network for one square meter is approximately 7.4 s when using a GTX 1080 GPU graphics card (Nvidia Corporation, 2016). By contrast, manually counting the flowers within a survey plot can take between 1 and 10 min, depending on the flower density. The predictions of the network have to be controlled by a good botany expert.

#### 4.3.2. Practical Considerations

Our main test grassland site was around 30 m by 30 m in size. To have enough overlapping images to generate an orthomosaic of this area with a sufficient ground sampling distance, a drone has to fly over the meadow for about 20 min. This requirement means that it is difficult to scale this approach to larger areas. A way of overcoming this problem would be to take images with less or no overlap at specific locations and to omit the generation of an orthomosaic. Knowing the flight height and the lens angle of the camera, one can calculate the covered area of the image. Running the prediction algorithm on these sample images would correspond to a stratified or random sampling of a larger area. This approach would also allow a higher flight height. With a longer focal length and eventually with a higher-resolution camera, the same ground sampling distance could be obtained while lowering the chance for potential disturbances of wildlife.

The advantage of the automated over the manual flower abundance determination approach is that much larger sample size can be collected. The effort to collect the vegetation data is smaller and more precise. This efficiency allows spending more time on controlling, extrapolating, and analyzing the data, which finally yields a better result. What remains to be evaluated is whether the prediction algorithm generates similar results close to the edges of an image as compared with the center. The viewing angle changes across an image and thus alters the appearance of the imaged objects (Aasen, 2016; Aasen and Bolten, 2018; Roth et al., 2018a). Consequently, there could be a degradation in prediction performance. The orthomosaics are created from only the center regions of the single images.

Various metrics are used to describe a model's performance. Precision, recall, F1 score, and mAP all describe a certain aspect of a model's performance. It depends on the application case which metric is most important. Precision and recall can easily be controlled with the minimum confidence parameter. The higher the minimum confidence parameter of the prediction script is set, the higher the precision becomes. Lowering the minimum confidence score increases the recall. For an abundance determination use-case as in this study, a balanced precision-to-recall ratio is advantageous because FN and FP predictions are likely to cancel each other out, and therefore a good estimate of the abundance can be given. The F1 score is mainly determined by precision and recall. The higher these two values are, the higher is the F1 score. A balanced precision-to-recall ratio improves the F1 score even more. Consequently, the F1 score is a good indicator of a model's performance.

We found that phenology impacted the results and that the model did not generalize well when flowers went into senescence. Besides, training a model with images from different ground sampling distances did not yield good performance. These findings suggest that the model does not generalize well over different sizes of the same flower and that keeping the ground sampling distance close to constant is important. However, when we trained the model for different ground sampling distances, the model worked well for a decrease in ground sampling distance down to 5 mm per pixel (Section 4.5; Figure 7). Still, the effect depends on the size of the flower, as shown by the example of *Lotus corniculatus*, for which the performance decreased significantly faster than for the larger flowers of *Leucanthemum vulgare* and *Knautia arvensis*. In the future, the results should be validated in more ways, e.g., by using cross-validation or by testing the models on more unseen test sites as well as including data with different environmental conditions.

The method developed in this study opens a wide range of use cases beyond the substitution of manual flower counting. Weed control could be realized in a precision agriculture setting. Detecting invasive neophyte plants in difficult-to-access areas could replace manual checks. The multi-temporal abundance maps have the potential to map flowering dynamics quantitatively and spatially, to assess co-occurrence of different flower species, and to assess the influence of climate conditions of different years on the abundance. By detecting certain indicator species, conclusions may be drawn about the soil properties. For example, the presence of *Leucanthemum vulgare* is an indicator of nutrient-poor meadows. In the context of quality assessment of meadows in connection with direct payments by the state, drone usage is imaginable. Apart from flowering plant detection, the method can be applied to other areas such as monitoring of wildlife aggregations as described by Lyons et al. (2019).

For some use cases, it might be beneficial to have real-time detections. The method developed in this study is not designed for that. By using the default configuration of the Faster R-CNN architecture without upscaling the images, the prediction algorithm can be sped up by a factor of four (at least). The drawback is that the accuracy is reduced with increased speed. Nevertheless, for some use cases, this reduction in accuracy might be acceptable. Using a lighter-weight object detection network design such as the single-shot detector architecture (Liu et al., 2016) can deliver further speed-ups. However, the accuracy is expected to be lower than with Faster R-CNN.

More training data would have been beneficial to better train the model on underrepresented flowers and catch flowers during their entire phenology. Unfortunately, this was not possible due to the failure of the initially used drone. However, with the now designed framework, new training data can be created and pooled with the current training data to expand the training dataset and allow better predictions in the future. The suite of tools developed in this study is easy to install and can be applied to any sort of object detection problem on aerial images. The time-consuming task of collecting training data by annotating aerial images can be carried out on the FieldAnnotator application for Android or with the widely used LabelMe application for desktop operating systems. The script that copies annotations onto overlapping images can be a powerful way of increasing the amount of training data without major efforts.

### 4.4. Very-High Resolution Remote Sensing and Deep Learning as a New Tool for Biodiversity Monitoring

Plant diversity can be estimated on different scales and granularity—from space-borne sensors down to *in-situ* measurements (Lausch et al., 2016, 2020; Wang and Gamon, 2019). Remote sensing based approaches cannot offer the same number of measurable traits as in-site measurements (Homolov et al., 2013). On the other hand, remote sensing allows mapping traits spatially explicit on larger scales. However, most often these traits are mapped on *via* proxies such as spectral data. For species identification, this brings some uncertainty since—in particular for objects relatively small to the GSD—the signature of a species can easily be diluted. With the advent of very- and ultra-high resolution remote sensing approaches (Aasen and Roth, 2016; Aasen et al., 2018a) in combination with deep learning, objects can now directly be identified and classified within the data based on their spatial and spectral features. As shown in this study, such approaches hold great promise for diversity monitoring.

Common deep learning network architectures are built to be feed with three band data (commonly RGB). First approach now also uses other spectral bands and even 3D information (Nezami et al., 2020) and we expect that these approaches will become more common when more very-high resolution spectral data is available.

## Data Availability Statement

The original contributions presented in the study are included in the article/Supplementary Material, further inquiries can be directed to the corresponding author.

## Author Contributions

HA, JG, MA, and BS conceived the ideas and designed the methodology. JG together with KJ from Agroscope, collected the data. JW was responsible for the drone flights and the Agisoft image processing. JG analyzed the data, programmed the software, and led the writing of the manuscript. HA led the research project, substantially contributed to the writing and revisions. All authors contributed to the drafts and gave final approval for publication.

## Funding

This research was partially funded by the Swiss National Science Foundation (SNSF) under the grant number 6571210, the project “PhenomEn” (IZCOZ0_198091) and within the framework of the National Research Programme “Sustainable Economy: resource-friendly, future-oriented, and innovative” (NRP 73), in the InnoFarm project, grant number 407340_172433. Moreover, it was carried out in the context of the Knowledge project of Agroscope (contract-ID: 655017678).

## Conflict of Interest

The authors declare that the research was conducted in the absence of any commercial or financial relationships that could be construed as a potential conflict of interest.

## Publisher's Note

All claims expressed in this article are solely those of the authors and do not necessarily represent those of their affiliated organizations, or those of the publisher, the editors and the reviewers. Any product that may be evaluated in this article, or claim that may be made by its manufacturer, is not guaranteed or endorsed by the publisher.

## Footnotes

^1^jittering refers to random changes in some parameters. For an illustrative overview of data augmentation one may refer to https://towardsdatascience.com/tagged/data-augmentation.
